# Toxico-pathological investigations of *in-ovo* inoculation of different fungal extracts and *Bacillus cereus* alone and in combination in chicken embryos

**DOI:** 10.5455/javar.2025.l997

**Published:** 2025-12-25

**Authors:** Qasim Saleem Raza, Muhammad Kashif Saleemi, Aisha Khatoon, Rao Zahid Abbas

**Affiliations:** 1 Department of Pathology, Faculty of Veterinary Science, University of Agriculture Faisalabad, Faisalabad, Pakistan; 2 Department of Parasitology, Faculty of Veterinary Science, University of Agriculture Faisalabad, Faisalabad, Pakistan

**Keywords:** Aflatoxin, *Bacillus cereus*, chick embryo, malformations, ochratoxin

## Abstract

**Objective:**

The current study was carried out to investigate the toxico-pathological and teratogenic effects of *in-ovo* administration of fungal-derived extracts of Ochratoxin (OT) and Aflatoxin (AF) and *Bacillus cereus* isolated from poultry feeds.

**Materials and Methods:**

Fertilized chicken eggs were divided into seven groups: control, sham control (normal saline), OT (600 ppb), AF (400 ppb), OT+AF (600 + 400 ppb), *B*. *cereus* (1 × 10⁸ CFU), and OT + AF + *B*. *cereus* (600 + 400 ppb + 1×10⁸ CFU). The extracts of each fungus and *B*. *cereus*wereinjected through the Chorioallantoic membrane route into 9-day-old embryos (216 h). The study evaluated embryonic mortality, hatchability, body weight, relative organ weights, and gross lesions. Morphometric alterations, including crown-to-rump, shank, head, and limb lengths, were measured.

**Results:**

Variable degrees of mortality and reduced hatchability were observed across treatment groups. Embryonic mortality was highest in combination groups F and G at 24 and 96 h, whereas the OT group showed the highest mortality at 48 and 72 h. Body weights and all morphometric parameters decreased significantly in the treated groups compared to the control groups. Teratogenic effects included curling, dwarfism, hemorrhages, stunted growth, feather loss, anophthalmia, malformed bills, twisted necks, abdominal hernias, and malformed fingers and limb buds.

**Conclusion:**

These findings suggest that inoculation of OT, AF, and *B*. *cereus*, individually or in combination, exerts severe teratogenic and embryotoxic effects, resulting in high embryonic mortality and developmental malformations.

## Introduction

Poultry farming is one of the fastest-growing sectors of the Pakistani economy, as it is a key contributor to the national gross domestic product. Avian health is critically dependent on the quality of poultry feed, which is negatively affected by the occurrence of mycotoxins. Such pollutants cause significant economic losses in the poultry sector, partly due to the high costs of feed production. In Pakistan, the prevalence of aflatoxins (AFs) in bird feed is high, with approximately 61% of the tested feed samples in 2011 being positive for mycotoxins [1].

Small organic compounds known as mycotoxins, which can contaminate a variety of agricultural products, including cereal grains (such as maize), coffee beans, nuts, soybeans, and spices, are produced by specific fungus species in the form of secondary metabolites. The most common mycotoxins in cereal crops are AFs, ochratoxin A (OTA), citrinin, patulin, trichothecenes, fumonisins, and zearalenone [1,2]. An analysis by the Food and Agriculture Organization of the United Nations has revealed that, worldwide, at least 25% of crop products are contaminated with mycotoxins. Approximately 2% of the nutritional and economic value of feed can be lost due to the presence of mycotoxins [3].

AFs are produced by various *Aspergillus* species, including *parasiticus* and *flavus* [1,4]. It is among the most common ones in poultry feeds and causes aflatoxicosis in various periods of production [3]. AFs B1, B2, G1, and G2 are naturally formed, but M1 and M2 are metabolites of AFB1 and AFB2, respectively, found in milk, cheese, eggs, etc. AFs are genotoxic & cytotoxic. According to the International Agency for Research on Cancer (IARC), AFs are categorized in Group 1 and are toxic to humans due to their teratogenic properties [5-7]. The occurrence of AFs in eggs, including embryonated eggs and feed, causes financial harm due to its impact on embryonic development and hatchability, as well as its role in numerous organ malformations [8]. It has been studied in different research papers that AFs have the ability to cause a higher number of mortalities in embryos due to their toxic effects. It also contributes to the loss of some functions of the membrane, like the loss of the intermediate membrane, cuticle, and eggshell membrane, i.e., internal [9,10].

There are three different types of ochratoxins (OTs), which are named as OTA, OTB, and OTC. *Aspergillus niger, Aspergillus ochraceous, Penicillium verrucosum,* and *Aspergillus carbonarius* are responsible for producing OTA, which is poisonous in nature. These OTs are produced by the species of *Penicillium* and *Aspergillus*. OTA is classified by the IARC as Group 2B, which indicates that it may cause human cancer [11,12]. The generation of reactive oxygen species, which prevents protein synthesis, lipid peroxidation, and DNA damage, is an OTA-mediated oxidative damage effect that is the most damaging to animal cells. In rats, rabbits, and chickens, OTA has been shown to be teratogenic and embryotoxic, both alone and in combination with other mycotoxins [13]. The presence of OTA, a mycotoxin known for its nephrotoxic effects, has been detected in poultry feed and its ingredients worldwide, including Pakistan [14]. In experimental trials, residues of OTA have been found in blood and tissues and in eggs [11,14] of those birds fed an OTA-contaminated diet.

*Bacillus cereus* is known to cause contamination of poultry from feed, infected chicks, dusty housing conditions, and industrial breeding. It is the probability that food and feed products contain sources of *B. cereus*, as some common commodities, such as meat, vegetables, wheat, and wheat products, can be positive for *B*. *cereus* [15]. *Bacillus* *cereus* also contributes to the gizzard erosion and ulceration syndrome in chicks [16,17].

The occurrence of mycotoxins and *B*. *cereus*, as well as their toxic responses in developing chick embryos, has led us to assess the toxico-pathological characteristics of indigenous cultures of fungi and *B. cereus* isolated from poultry feeds. There is a lack of data about the toxic effects of *B*. *cereus* in embryonated eggs. Therefore, the current study aimed to illustrate the different toxic effects and harmful results of fungal extracts and *B. cereus*, which are isolated from poultry feed, on the development of embryonated chickens.

## Materials and Methods

### Ethical approval

This study received ethical clearance from the Institutional Biosafety and Bioethics Committee (IBC) of the University of Agriculture Faisalabad, as per letter No. 731/ORIC dated 20 February 2024.

### Dose formulation

Doses of AF, OT, and *B. cereus* were prepared in the Avian Molecular and Toxicologic Pathology Laboratory, Department of Pathology, Faculty of Veterinary Science, University of Agriculture Faisalabad. AF and OT were procured from NRRL 6540 & CECT 2687, as well as link Fries. A (CECT: 2948), Culture Collection Center, University of Valencia, Spain, respectively, and stored in the Department of Pathology, Faculty of Veterinary Science, UAF. Poultry feed samples were collected from various poultry farms and then added to Tryptone Soy Broth (TSB) for the isolation of *B. cereus*. A loop full of broth culture was streaked onto selective mannitol egg-yolk polymyxin (MYP) agar, and the plates were incubated at 35°C–37°C for 24 h. The eosin-pink-colored colonies on MYPA were identified to be *B*. *cereus,* and to identify toxin-producing genes, a polymerase chain reaction was performed.The concentrations of AF and OT were estimated by high-performance liquid chromatography [18]. The calculated amount of AF, OT, and *B*. *cereus* was 400 ppb, 600 ppb, and 1 × 10^8^ CFU per vial, respectively [19,20]. AFB1 and OT filtrate were mixed with 99.9% absolute ethanol, and their concentration was brought to 10% through an 8.5% saline solution to make different dose rates of AF and OT [21].

### Experimental framework

#### Study/dosage groups and pre-hatch exposure

A total of 70 fertilized eggs of *Gallus domesticus* were taken from the broiler breeder flock, & these eggs were allocated into seven groups: Group (A) was the control, Group (B) was the sham control, Group (C) was given AF (400 ppb), Group (D) was treated with OT (600 ppb), Group (E) was treated with *B*. *cereus* (1×10^8^ CFU), Group (F) was treated with AF and OT (400 + 600 ppb), and Group (G) was treated with AF, OT,and* B*. *cereus* (400 + 600 ppb + 1×10^8^ CFU). Each group contains 10 eggs. For propagation of *B*. *cereus*, TSB broth was used. Before injecting the solutions, the eggs were cleansed and disinfected with 70% alcohol. The test solutions were then injected into the eggs through the Chorioallantoic membrane immediately before the eggs were placed in the incubator. The test solution was introduced into the air sac after piercing the shell of the egg at the blunted tips of the eggs [20]. Syringes were employed for injecting 0.1 ml of fluid. Following the inoculations, the holes were sealed with paraffin wax, and the eggs were placed in an incubator set at 37.5°C and a relative humidity of 52%.

### Parameters studied

#### Mortality

The percentage mortality was calculated by recording the number of embryos lost throughout the incubation period. The incubation period was 21 days from laying day until hatch. The incubation temperature was maintained at 37.5°C with a relative humidity of 65%.

#### Body weights

On the day of hatching, the body weights of each embryo were calculated.

#### Morphometric measurements

The weight of embryos, crown-rump length, head length, and foot and shank lengths were measured and recorded.

#### Data analysis

For statistical analysis, one-way ANOVA was employed, and group means were compared using the DMR-test in MSTATC statistical software.

## Results

### Mortality rates

**Figure 1.**
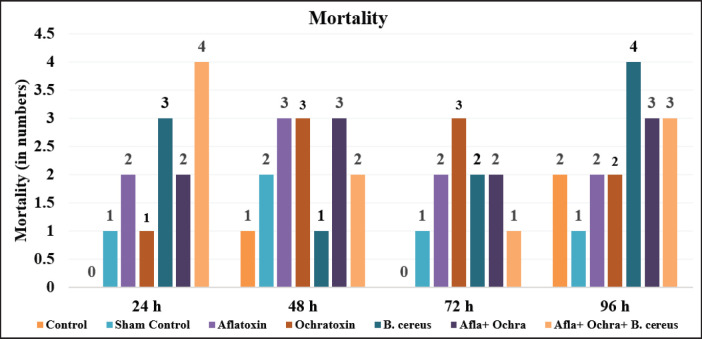
Graph showing mortality rate in different groups.

Daily mortality was computed by recording the number of embryos that died throughout the incubation. The death rate was high in the combination group, which was administered with extracts of AF, OT, and *B*. *cereus.* At 48 h of inoculation, mortality was high in AF, OT, and combined toxin groups. After 96 h, the highest mortality was seen in the *B.* *cereus* group. At 72 h post-infection, the mortality was high in the OT group as compared to all other groups (Table 1 , Fig. 1).

**Table 1.** Mortality of chick embryos administered different types of toxins and*B*.*cereus*(*n*= 10 embryos per group).

Groups	24 h	48 h	72 h	96 h	Overall mortality	Mortality (%)
Control	0	1	0	2	3	30%
Sham control	1	2	1	1	5	50%
AF	2	3	2	2	9	90%
OT	1	3	3	2	9	90%
*Bacillus cereus*	3	1	2	4	10	100%
AF + OT	2	3	2	3	10	100%
AF + OT + *Bacillus cereus*	4	2	1	3	10	100%

Values (Mean + SD) differ significantly (*p* ≤ 0.05). (*n* = 10/group).

### Body weights

**Figure 2.**
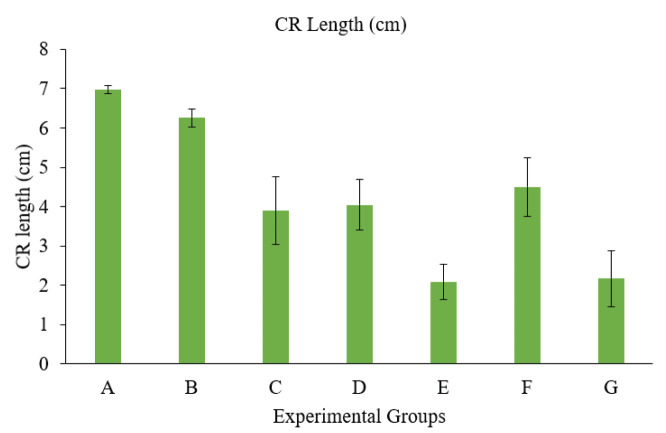
Graph showing the weight of embryos in different Groups. Group A = Control; Group B = Sham control; Group C = AF (400 ppb); Group D = OT (600 ppb); Group E = *Bacillus cereus* (1 × 10^8^ CFU); Group F = AF + OT; Group G = AF + OT + *Bacillus cereus.*

All the treatment groups had lower body weights in the embryos that hatched from eggs given different doses of toxins and *B. cereus* compared to the controls. Body weights have been presented in Table 2 and Figure 2.

**Table 2.** Effect of different toxins and*B. cereus*on the body weight and different lengths of body parts of chick embryo after hatching (Means ± SD) (*n*= 10 embryos per group).

Groups	Body weight (gm)	CR length (cm)	Shank (cm)	Head (cm)	Toe (cm)
A	19.796 ± 0.79^a^	6.973 ± 0.11^a^	1.081 ± 0.10^a^	2.068 ± 0.08^a^	1.809 ± 0.08^a^
B	18.026 ± 1.76^b^	6.257 ± 0.23^a^	1.165 ± 0.11^a^	1.794 ± 0.17^b^	1.506 ± 0.14^a^
C	4.344 ± 0.76^c^	3.899 ± 0.87^b^	0.456 ± 0.22^c^	1.257 ± 0.14^c^	0.311 ± 0.12^a^
D	4.108 ± 0.58^cd^	4.041 ± 0.64^b^	0.541 ± 0.16^c^	1.200 ± 0.33^c^	0.272 ± 0.04^a^
E	2.930 ± 0.45^d^	2.087 ± 0.44^c^	0.218 ± 0.08^d^	0.583 ± 0.14^d^	0.226 ± 0.07^a^
F	4.020 ± 0.48^cd^	4.500 ± 0.74^b^	0.890 ± 0.11^b^	1.227 ± 0.22^c^	1.586 ± 0.21^a^
G	3.280 ± 0.45^cd^	2.175 ± 0.71^c^	0.752 ± 0.07^b^	0.824 ± 0.14^d^	0.142 ± 0.06^a^

Values (Mean + SD) differ significantly (*p* ≤ 0.05). Group A = Control, Group B = Sham Control, Group C = AF (400 ppb) Group D = OT (600 ppb), Group E = *Bacillus cereus* (1 × 10^8^ CFU), Group F = AF + OT, Group G = AF + OT + *Bacillus cereus*.

### Morphometric alteration measurements

**Figure 3.**
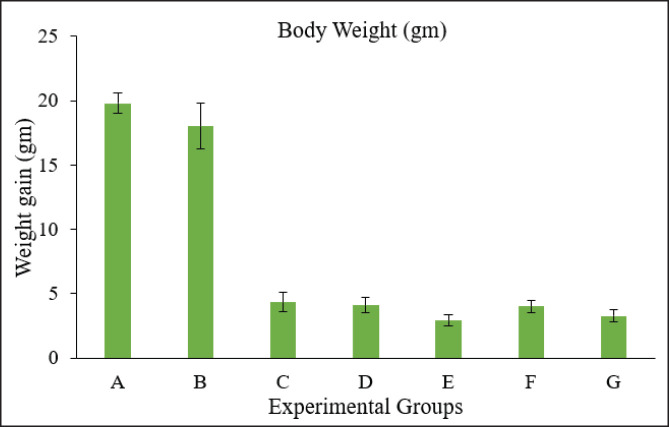
Graph showing the weight of embryos in different Groups. Group A = Control; Group B = Sham control; Group C = AF (400 ppb); Group D = OT (600 ppb); Group E = *Bacillus cereus* (1 × 10^8^ CFU); Group F = AF + OT; Group G = AF + OT + *Bacillus cereus.*

**Figure 4.**
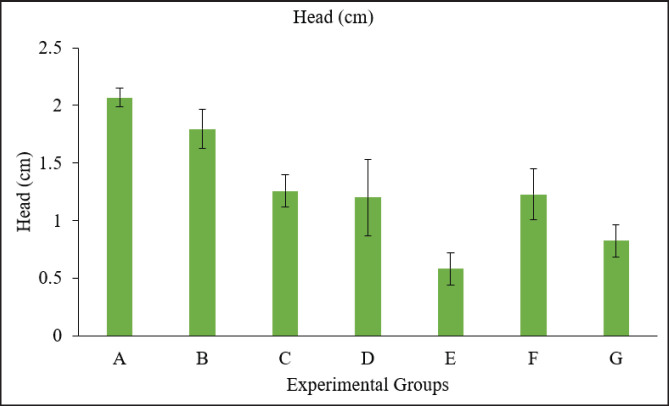
Graph showing Shank length of embryos in different groups. Group A = Control; Group B = Sham control; Group C = AF (400 ppb); Group D = OT (600 ppb); Group E = *Bacillus cereus* (1 × 10^8^ CFU); Group F = AF + OT; Group G = AF + OT + *Bacillus cereus.*

**Figure 5.**
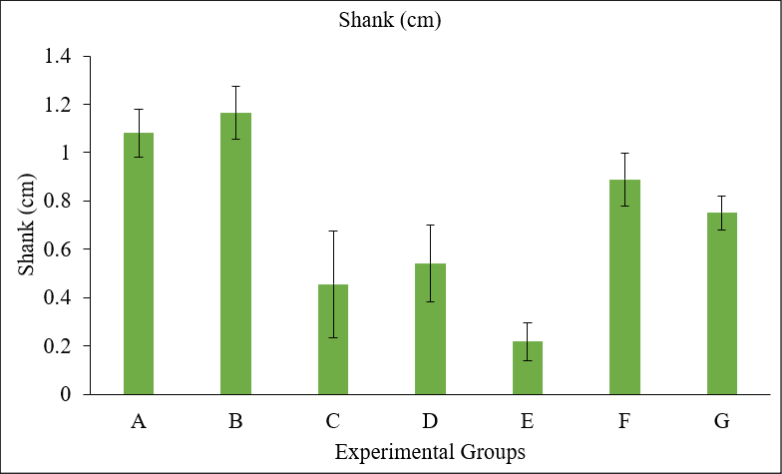
Graph showing the head diameter of embryos in different groups. Group A = Control; Group B = Sham control; Group C = AF (400 ppb); Group D = OT (600 ppb); Group E = *Bacillus cereus* (1 × 10^8^ CFU); Group F = AF + OT; Group G = AF + OT + *Bacillus cereus*.

**Figure 6.**
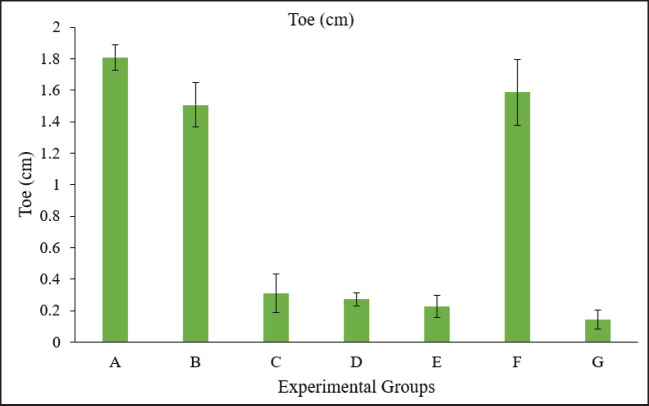
Graph showing the toe length of embryos in different groups. Group A = Control; Group B = Sham control; Group C = AF (400 ppb); Group D = OT (600 ppb); Group E = *Bacillus cereus* (1 × 10^8^ CFU); Group F = AF + OT; Group G = AF + OT + *Bacillus cereus*.

The crown-to-rump length was calculated from the top of the head to the bottom of the embryo. There was a higher degree of reduction in length from crown to rump in the groups treated with toxins compared to the control group, whereas no significant difference was observed among the treated groups (Fig. 3). A Vernier caliper was used to measure the length of the head from anterior to posterior, i.e., from the point of the beak to the occipital bone. A significant reduction in various measurements of head circumference was observed, with no significant difference among the treatment groups (Fig. 5). A significant decrease in shank length was observed in the treated group compared to the control group, whereas no significant differences were observed among the treated groups (Fig. 4). The highest toe size was observed in the control group, and no significant difference was noted among the treated groups (Table 2, Fig. 6).

### Congenital malformations/abnormalities

**Figure 7.**
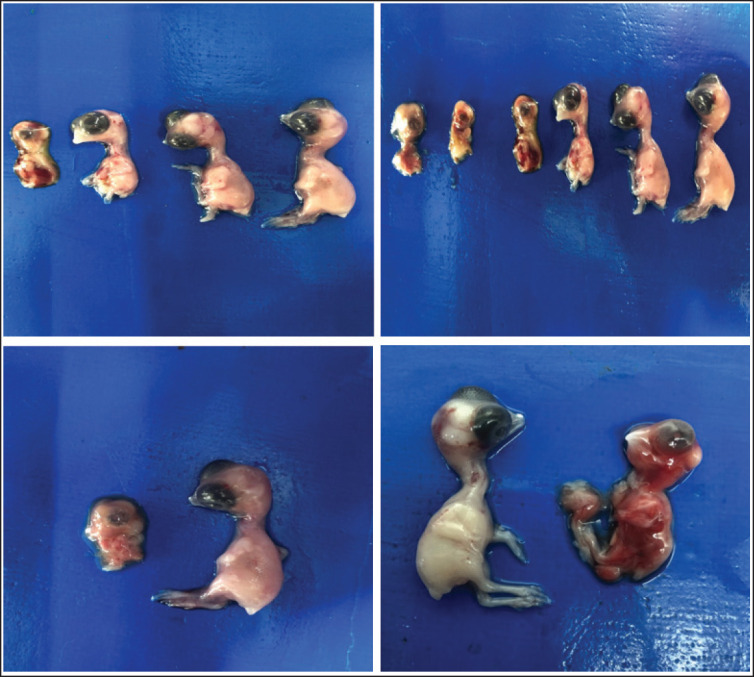
Photographs of chick embryos treated with toxins and *B*. *cereus* showing growth retardation, highly deformed head, anophthalmia, absence of feathers, long neck, abdominal hernia, and deformed limbs.

**Figure 8.**
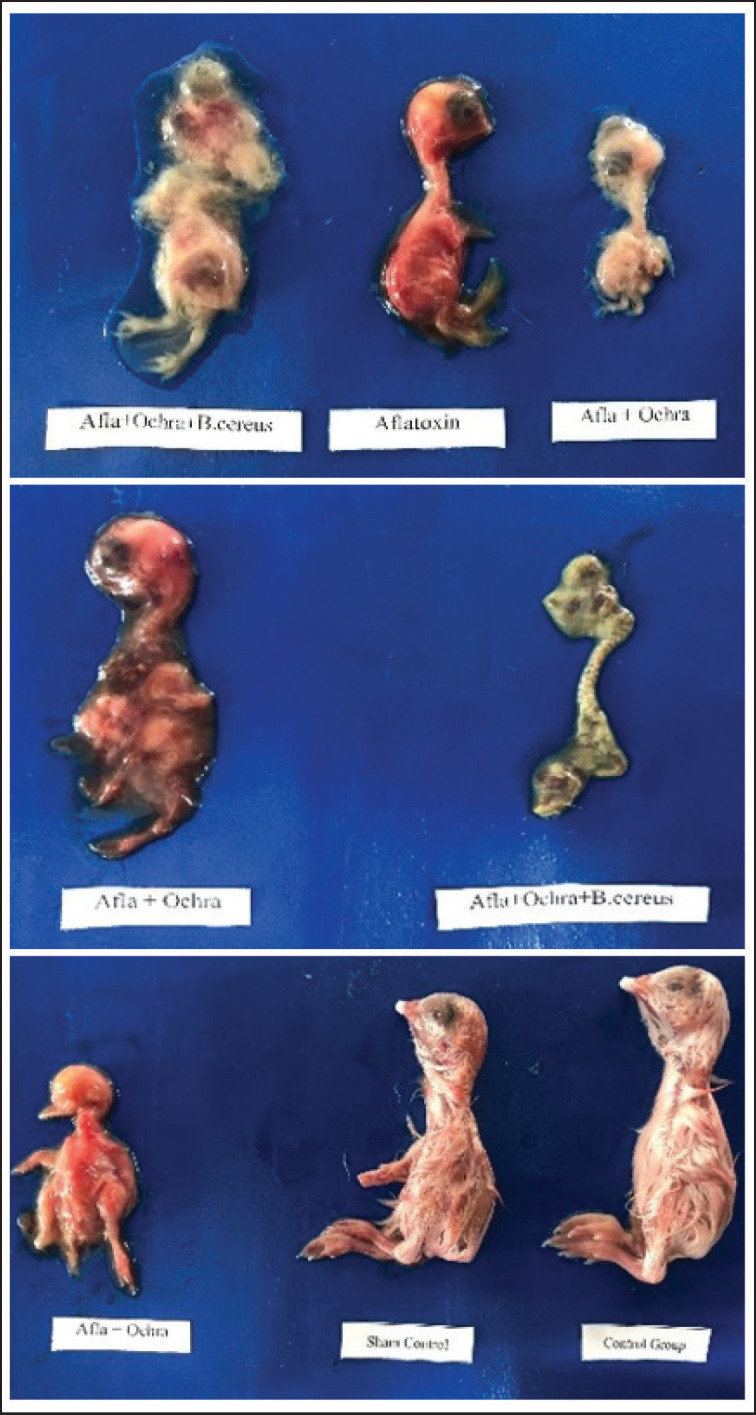
Photographs of chick embryos treated with different fungal extracts and *B*. *cereus* showing growth retardation, deformed head, invaginated eyes, absence of feathers, twisted neck, abdominal hernia, deformed wings and digits, reversed orientation of the legs, and deformed toes. Hemorrhages and dwarfism are shown in middle Figure 8.

Developmental defects of embryos were assessed. Based on physical body parameters, some of the effects were evaluated. The abnormalities in different groups included hemorrhages, dwarfism, growth impedance, loss/nonappearance of feathers, various head deformities, anophthalmia, invaginated eyes, malformed beaks, wry or long necks, abdominal hernias, and malformed fingers, toes, and bud limbs. Such developmental defects are obvious in gross images (Figs. 7, 8).

## Discussion

According to the current study, the death rates in all the treated groups were greater than those in the control group. 100% mortality was observed in the*B. cereus*group (E) and in those combined (F and G). Ali et al. [21] also reported the highest mortality in the AFB1-treated group inoculated into embryonated eggs. According to Zuo et al. [22], when AFB1 is administered *in ovo* to developing embryos, there were notable fatalities, abnormalities in the developing embryo, and the hatching of chicks with weak immune systems. The current study’s higher mortality and lower hatchability rates align with the findings of Lizárraga-Paulín et al. [23], who demonstrated that broiler chickens fed an AF-rich diet performed worse than controls in terms of growth, performance, and survival rate, as well as exhibited higher mortality rates. The findings supported the effect of OTA on both initial and late embryo death, with chick hatchability declining as dose rates of toxin increased. Liu et al. [24] have previously confirmed the teratogenic effects of OTA in *in-ovo* testing at different doses; our results were also in line with [18,24]. Bryła et al. [25] also studied the same effects of OT in an *in-ovo* experiment. The two types of AF-induced embryonic deaths are (i) early embryonic deaths, which are typically caused by the direct cytotoxic effects of high doses of toxins, and (ii) late deaths, which are connected to the metabolism of AF in the embryonic liver and result in the production of extremely toxic metabolites [25,26].

In our study, the body weights of embryos were also significantly lower in the treatment groups, i.e., toxin-treated eggs, compared to the control and sham control groups. Weight and morphometric measurements of different parts of the body in those groups receiving 5, 10, and 20 ng of AFB1 were considerably worse in contradiction to the control, which showed a poorer growth rate than normal, as observed by Saleemi et al. [20]. The weight of the hatched chick, as demonstrated by the authors, was several times less than that of the control group. However, this was only apparent in the groups receiving the highest dosage of OTA *in ovo* [27]. This reduction in embryo body weight is attributed to the detrimental effects of OTA, primarily a decline in protein synthesis, which is crucial for embryo growth. Zuo et al. [22] showed similar results, in which hatched chicks in the AF-administered group had significantly lower body weight. The hepatotoxic impact of the toxin may have contributed to the decrease in weight of the birds that hatched from the AFB1-injected eggs.

The morphological abnormalities, including hemorrhages, dwarfism, growth impairment, loss or absence of feathers, various cranial deformities, anophthalmia, invaginated eyes, malformed beaks, wry or elongated necks, abdominal hernias, and malformations of fingers, toes, and bud limbs, were recorded in this study. This can be explained by the altering effects of mycotoxins and *B. cereus*. Our findings are corroborated by Zuo et al. [22]. Liu et al. [24] and Saleemi et al. [20] report on some embryonic malformations, including reduced body size, mandibular hypoplasia, anophthalmia, and maxillary retrognathism. Numerous research studies have verified the embryotoxic potential of OTA, which has been thoroughly investigated [19,24,25,27-29].

The survival and hatchability of the embryo can be negatively impacted by the residual AF in eggs, and it may even cause organ abnormalities [30,31]. The teratogenic effects of OTA observed by Liu et al. [24] were similar to those in our study. As Bryła et al. [25] have previously demonstrated, these effects of OTA may be caused by the creation of DNA adducts and consequent suppression of protein synthesis.

This study has several limitations that should be acknowledged. First, only a single dose of *B. cereus* or other toxins was tested, which restricts the ability to evaluate dose-dependent effects. Second, the assessment of toxicity and developmental impact was based solely on external observations, such as mortality, hatching success, and gross malformations. No histopathological examinations were performed on internal organs or tissues, which limits the ability to detect subtle or microscopic changes that may have occurred without visible signs.

## Conclusion

Inevitably, no trustworthy report about the impact of *B*. *cereus* on the developing chick embryo exists in the published accessible literature, and to the best of our knowledge, this study is the preliminary study for assessing the effects of *B*. *cereus* on chicken embryos. The available information about embryotoxic effects of OTA and AFB1 has been compared with our findings. The results of this investigation demonstrate the importance of assessing the toxigenic characteristics of local fungal isolates and *B*. *cereus*. Based on the findings of this preliminary investigation, the following recommendations are proposed: additional in-depth studies are necessary to fully understand the mechanisms underlying the embryotoxic effects of *B*. *cereus*, including dose-response relationships, time-dependent impacts, and potential synergistic effects with other microbial or chemical toxins. Furthermore, future research should focus on molecular characterization of *B*. *cereus* strains to distinguish between pathogenic and non-pathogenic isolates, particularly in the context of poultry production systems.

## List of abbreviations

AF, aflatoxin; AFB₁, aflatoxin B₁; ANOVA, analysis of variance; CAM, Chorioallantoic membrane; CECT, Colección Española de Cultivos Tipo (Spanish Type Culture Collection); CFU, colony forming unit; DMR, Duncan’s multiple range test; DNA, deoxyribonucleic acid; HPLC, high-performance liquid chromatography; IARC, International Agency for Research on Cancer; IBC, Institutional Biosafety and Bioethics Committee; MYP, mannitol egg-yolk polymyxin agar; NRRL, Northern Regional Research Laboratory; OT, ochratoxin; OTA, ochratoxin A; ppb, parts per billion; TSB, tryptone soya broth; h, hours.
